# The impact of diet, exercise, and sleep on *Helicobacter pylori* infection with different occupations: a cross-sectional study

**DOI:** 10.1186/s12879-024-09505-8

**Published:** 2024-07-11

**Authors:** Shiwen He, Xue He, Yinglong Duan, Yating Luo, Yuxuan Li, Jing Li, Ying Li, Pingting Yang, Yaqin Wang, Jianfei Xie, Min Liu, Andy SK Cheng

**Affiliations:** 1 Health Management Center, The Third Xiangya Hospital, Central South University, Changsha, Hunan China; 2 Nursing Department, The Third Xiangya Hospital, Central South University, No. 138, Tongzipo Road, PO Box 410013, Changsha, Hunan China; 3 School of Health Sciences, Western Sydney University, Sydney, Australia

**Keywords:** Diet, Exercise, Sleep, *Helicobacter pylori* Infection, Occupations, Cross-sectional study

## Abstract

**Background:**

Associations between *Helicobacter pylori* infection and lifestyle factors vary greatly by geographic location. This study aims to evaluate the prevalence of *Helicobacter pylori* infection in the Hunan cohort of central China and analyze the associations between *Helicobacter pylori* infection and lifestyle factors in different occupations.

**Methods:**

This was a cross-sectional study. Participants who received an annual physical examination were invited. *Helicobacter pylori* infection was detected by the 13 C-urea breath test. Self-reported physical examination questionnaires were used to analyze participants’ demographic information, diet, exercise status, and sleep situations.

**Results:**

23254 participants finished this study. The *Helicobacter pylori* infection rate in the Hunan area was 25.8%, with the lowest prevalence in students (8.5%) and the highest prevalence in business managers (29.9%). The risk factors for *Helicobacter pylori* infection were marital status (divorced or married) (OR:1.16, 95%CI:1.090–1.234), overeating (OR:1.105, 95%CI: 1.001–1.220), and consumption of eggs (OR:1.047, 95%CI:1.004–1.092), animal viscera (OR: 1.077, 95%CI:1.014–1.144) and coffee (OR:1.074, 95%CI:1.019–1.132). Participants’ education level (OR:0.911, 95%CI:0.881-0942), consumption of midnight snack (OR:0.926, 95%CI:0.877–0.977), and vegetable (OR:0.927, 95%CI: 0.884–0.972) were protective factors against *Helicobacter pylori* infection. Whether participants exercised regularly or had sleep problems had no significant effect on *Helicobacter pylori* infection. Different professionals showed significant differences in the rates of overeating, eating three meals on time, midnight snack, and consuming coffee, eggs, animal viscera, and vegetables > 3 times/week (P values < 0.05).

**Conclusions:**

*Helicobacter pylori* infection showed a significant relationship with dietary factors, but not significantly with sleep and exercise factors. Different occupations showed different dietary tendencies related to *Helicobacter pylori* infection. The design of an occupation-based *Helicobacter pylori* screening and prevention program is supported.

## Background

The WHO reported that malignancies caused by infectious agents (including *Helicobacter pylori*, *H. pylori*) accounted for 20% of malignancies in developing countries [1].* H. pylori* has been identified as a Class I carcinogen and as a major etiologic agent of infection-associated cancers, responsible for approximately 90% of gastric cancer cases [2]. *H. pylori *can lead to chronic gastritis, peptic ulcer, gastric adenocarcinoma, and mucosa-associated lymphoid tissue lymphoma and is associated with idiopathic thrombocytopenic purpura [3]. In addition, *H. pylori *may be associated with hepatobiliary, cardiovascular, and allergic diseases, and people with a medical history of cholecystitis/cholecystolithiasis, hypertension, and asthma had a significant negative association with *H. pylori* infection [4]. Thus, preventing *H. pylori* infection is important for preventing gastrointestinal-related diseases and extra gastric diseases.

*H. pylori* infection is highly prevalent worldwide, with a global prevalence of 44.3% in 2015–2022 [5]. *H. pylori* infection varied by location and sanitation standards [6], prevalence of *H. pylori *infection was 41% in the United Arab Emirates, 41.5% in Korea, 87.2% in Spain, and 84% in Portugal and Poland [7, 8]. According to a meta-analysis, the worldwide prevalence of *H. pylori* infection (50.8%) in developing countries are higher than in developed countries(34.7%) [9]. In China, the prevalence decreased from 58.3% in 1983–1994 to 40.0% in 2015–2019. The prevalence of *H. pylori* infection was high in the northwest (51.8%), east (47.7%), and southwest (46.6%) of China and low in the central region [10, 11]. Changsha city is in the central region of China, and the previous study focused on the Changsha population was limited to children [12], large sample size cross-sectional surveys are still needed.

Environmental factors is one of the three parts of main pathophysiology of *H. pylori* infection [6]. Previous studies have revealed that lifestyle, mostly diet, was associated with *H. pylori* infection, such as binge drinking, coffee, dairy food, vegetables, and fruit [13–16]. Exercise and sleep had some impact on *H. pylori*-related gastric disease [17–19]. The pathological mechanism may involve metabolic disorders associated with *H. pylori *infection, as conventional *H. pylori* eradication therapy promoted positive changes in glucose and lipid profiles [20]. Differently, Habbash and colleagues [15] reported that exercise and sleep had no significant impact on *Helicobacter pylori* infection in 200 Bahraini adults. Thus, the association between lifestyle (diet, exercise, and sleep) and *H. pylori* infection needs more evidence.

Lifestyle-associated variables (including exercise and diet) mediated the association between occupations and chronic diseases, such as cardiovascular disease and metabolic syndrome [21, 22]. People with different occupations had reported significant differences in *Helicobacter pylori* infection [4, 11]. The associations of *H. pylori* infection with lifestyle, diet, exercise, and sleep in different occupations were less reported, and the benefit of eradication at the occupational level has not yet been adequately studied [23]. Therefore, this study aimed to indicate the prevalence of *H. pylori* infection in the Hunan cohort of central China and analyze the associations of *H. pylori* infection with lifestyle, diet, exercise, and sleep in different occupations.

## Methods

### Study design and implementation

A cross-sectional study was carried out at the health management center of a general tertiary hospital located in the Hunan area in central China between January 1, 2015, and December 31, 2020. The health management center is one of the first health management flagship units in China (the only one in Changsha, Hunan area), which can receive over 800 people for physical examinations every day and has signed the health management services with tens of thousands of people from more than 20 communities and rural residential villages. Participants who received annual physical examinations were invited. For participants who were under the age of 18, consent from the guardian was needed. The exclusion criteria were as follows: (1) did not sign the consent form; (2) pregnancy preparation, pregnancy status, and breast-feeding status; and 3)undertaking proton pump inhibitors(PPI)/antibiotics/bismuth-containing compounds/anti-inflammatory drug within the past month (diseases such as bleeding from peptic ulcers, stomach surgery, gastric MALT lymphoma, severe atrophy of gastric mucosa).

Written informed consent was obtained from the two health management centers. With the help of the directors of health management centers, three trained study members (post-graduate students) recruited participants at the physical examination registration points and explained the aims, procedures, and risks of this study to participants. After participants agreed and signed the informed consent, one of the study members would send the questionnaire link to the participants’ mobile phones and guide them to complete the filling out. Participants were requested to fill out electronic questionnaires within 24 h. The questionnaire links were formed by the Self-rated Physical Examination Questionnaire System (website: https://new.selfhealth.cn, patent number CN201710710888.1). Then, participants received physical examinations and *H. pylori* infection tests. 23,254 participants finished the study, and the response rate was 74.3%.

### Measurements

*H. pylori* was detected by 13 C-UBT detection. The demographic information, diet, exercise, and sleep situations of participants were investigated by Self-rated Physical Examination Questionnaires.

### 13 C-UBT detection

13 C-UBT detection is one of the general methods to detect *Helicobacter pylori* in the stomach. 13 C-UBT detection was regarded as positive when the 13 C radioactive signal values (delta-over-baseline, DOB) were greater than or equal to 4. Before detection, participants fasted for at least 2 h and exhaled CO_2_ was collected using a special gas cylinder. Then, participants consumed the 13 C capsule with cold drinking water. After 20 min, the exhaled CO_2_ was collected using a special gas cylinder and measured by an *H. pylori* detector. Drugs such as PPIs, antibiotics, bismuth agents, and gastric mucosa protectors will affect the activity of urease, so patients are required to stop using antibiotics, bismuth compounds, and other drugs for at least 4 weeks and PPI and sucralfate for at least 2 weeks before testing [24].

### Self-rated physical examination questionnaire

The self-rated Physical Examination Questionnaire was published by the Chinese Medical Association in 2014, aiming to screen chronic diseases and related risk factors [25], and formed into an electronic system (the Self-Rated Physical Examination Questionnaire System) in 2017. The self-rated physical examination questionnaire collected six dimensions of subjective health information (health-related history, physical symptoms, lifestyle and environment, mental health and mental stress, sleep health, and health literacy) [25], with the coefficient of Cronbach α ranged from 0.669 to 0.917, the split-half reliability ranged from 0.572 to 0.877, and the test-retest reliability ranged from 0.693 to 0.896 [26]. The questionnaire was widely used and can improve the detection rate of risk stratification of patients with chronic diseases (such as hypertension) [27]. This study analyzed the data on participants’ demographic information, lifestyle, and sleep health. The content is as follows:

### Demographic information

Participants’ demographic information was collected, including age, gender, body mass index (BMI), marital status, education level, medical insurance, chronic disease, long-term medication, and occupation.

### Lifestyle

Dietary habits were assessed: (1) eating patterns, including staple food, food taste preference, eating three meals on time, midnight snacks, and overeating. Eating three meals on time was defined as having breakfast between 7:00–9:00, lunch between 11:00–13:00, and dinner between 17:00–19:00. Midnight snacks were defined as snacks between 22:00 and 04:00. Overeating was defined as binge eating and drinking without restraint or regularity occurring at least 3 times within 6 months. The frequency of eating three meals on time and midnight snacks was valued by “<2 times/week”, “2 ~ 3 times/week” and “>3 times/week”. (2) Food categories: sugar-sweetened beverages, coffee, and alcohol; milk and dairy products, eggs, beans, or beans products (beans, nuts, and seeds), fruit, vegetables, livestock meat (pork, poultry, beef, and organs), oil and fat, animal viscera, fish and shrimp (seafood, freshwater fish and aquatic products). The frequency of drinking and food intake categories were valued by “never”, “seldom (1 ~ 2 times/week)”, “sometimes (3 ~ 5 times/week)” and “often (> 5 times/week)”.

The exercise was assessed. Regular exercise was defined as exercising during leisure time at least once per week. Years of exercising regularly, frequency of exercising, and sustained exercise time per day were evaluated.

### Sleep health

Night sleeping time and sleep problems were evaluated. Sleep problems were defined as having had sleep problems at least 3 times per week. The four types of sleep problems included difficulty falling asleep (cannot fall asleep within 30 min), early waking, excessive dreaming or waking from a nightmare, and nighttime awakening (waking up more than twice after falling asleep).

### Sample size estimation

According to previous data [11], it is found that the pooled prevalence of* H. pylori* infection in chinese mainland was 43.7%. We required that with 95% conffdence, the results need to fall within 10% of the overall truth rate. According to the calculation formula N = Z^2^_1−α/2_(1 − p)/ε^2^γ, it is calculated that *N* = 495. Considering the 10% of attrition rate and the design efffciency, the questionnaires required for this cross-sectional study were at least 1089 in total.

### Statistical analysis

SPSS 19.0 for Windows (IBM Corp, Armonk, NY, USA) was used for all statistical analyses. Participants’ age and BMI index were expressed as the mean ± standard deviation (SD), and categorical data such as sex, marital status, and education level were expressed as frequencies and percentages. Independent samples t-tests and χ^2^ tests were used to analyze the differences in *H. pylori* infection rates among populations with different diet, sleep, and exercise characteristics. Logistic regression models (stepwise, with the criteria for inclusion being 0.10 and for exclusion being 0.15) were used to select the optimal subset of independent variables in all participants. A P value < 0.05 was considered to indicate a significant association with *H. pylori* infection.

## Results

### Demographic characteristics of the participants

A total of 31,300 participants were invited to participate in this study. 8046 participants were excluded because of not signing the consent form, unfinished 13 C-UBT detection, and incomplete questionnaires. Finally, 23,254 participants were enrolled in this study.

The overall *H. pylori* infection rate in the Hunan area was 25.8%. Participants with *H. pylori* infection were 44.52 ± 10.865 years old and had a BMI of 25.07 ± 3.17. Participants who were female, married or divorced, had a lower educational background, and had a chronic disease, long-term medication use, and medical insurance showed a higher prevalence of *H. pylori* infection (details in Table 1).

**Table 1:** Correlation between the prevalence of *Helicobacter pylori* infection and demographic information

Variable	*H. pylori*-positive *N*(%)	*H. pylori*-negative *N*(%)	t/ x^2^	*P* values
Age	44.52 ± 10.865	44.18 ± 10.718	-2.063	0.038
Gender				
Male	5341(25.5)	15,570(74.5)	5.130	0.024
Female	649(27.7)	1694(72.3)		
BMI (kg/m^2^)	25.07 ± 3.17	24.93 ± 3.194	-2.817	0.005
Marital status				
Single	325(18.8)	1408(81.2)	50.630	< 0.001
Divorced	141(29.5)	337(70.5)		
Married	5524(26.3)	15,519(73.7)		
Education level				
Junior school or below	666(29.7)	75(70.3)	113.004	< 0.001
Technical secondary school	970(29.0)	2371(71.0)		
High school	2866(26.0)	8156(74.0)		
College or Undergraduate	504(27.6)	1323(72.4)		
Graduate degree and above	984(20.4)	3839(79.6)		
Medical insurance				
No	823(21.4)	3019(78.6)	59.385	<0.001
Yes	4733(27.0)	12,794(73.0)		
Unclear	434(23.0)	1451(77.0)		
Chronic disease				
No	3758(25.1)	11,189(74.9)	8.325	0.004
Yes	2232(26.9)	6075(73.1)		
Long-term medication				
No	4851(25.7)	14,060(74.3)	0.609	0.435
Yes	1139(26.2)	3204(73.8)		
Occupation				
Students	7(8.5)	75(91.5)	118.363	< 0.001
Unemployed	318(28.1)	813(71.9)		
Farmers	259(29.4)	623(70.6)		
Professional and technical personnel	1453(24.1)	4567(75.9)		
Militaries	1261(26.2)	3556(73.8)		
Business managers	1434(29.9)	3357(70.1)		
Factory workers	162(27.4)	429(72.6)		
Retired people	239(26.4)	667(73.6)		
Other	857(21.2)	3177(78.8)		

**Fig. 1:**
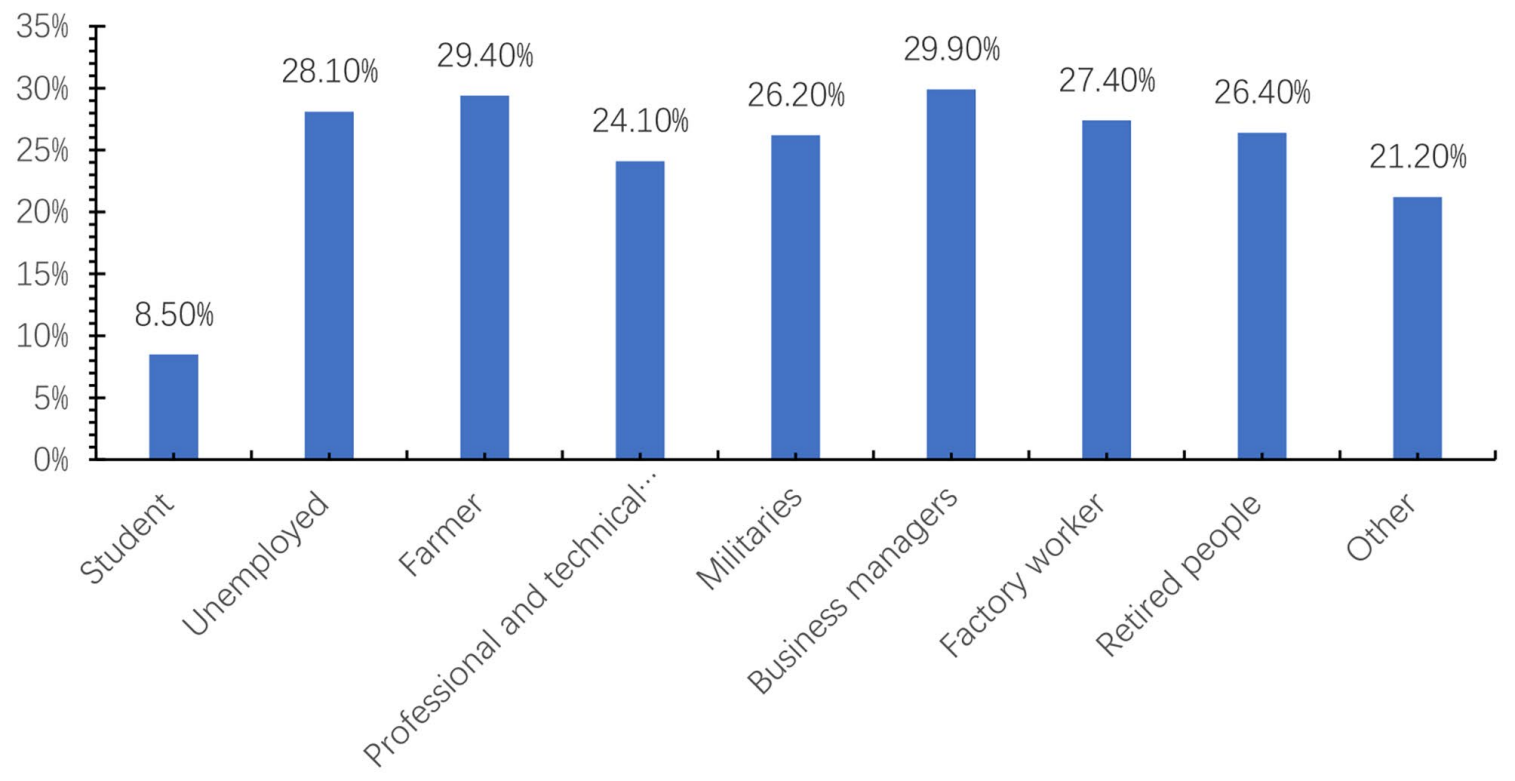
Percentage of *H. pylori* infection in different occupations

As shown in Fig. 1, the three occupations with the highest *H. pylori* infection prevalence were business managers, farmers, and unemployed individuals. Students and professional and technical personnel showed a relatively low prevalence of *H. pylori* infection.

### Prevalence of *Helicobacter pylori* infection and dietary habits

Participants who ate midnight snacks rarely (*P* = 0.004), overeat (*P* = 0.034), and drink coffee rarely or occasionally (*P* = 0.046) revealed a higher prevalence of *H. pylori* infection. Participants who had a low frequency of fruit *(P* = 0.031) and vegetable (*P* = 0.050) consumption and a high frequency of animal viscera consumption (*P* = 0.009) showed a significantly higher prevalence of *H. pylori* infection. Details are shown in Table 2.

**Table 2:** Correlation between the prevalence of *Helicobacter pylori* infection and dietary habit

variable	*H. pylori*-positive *N*(%)	*H. pylori*-negative *N*(%)	x^2^	*P* values
Eating pattern				
Staple food				
Mainly refined grains	2803(26.4)	7798(73.6)	5.379	0.146
Mainly coarse grains	429(25.5)	1256(74.5)		
Refined and coarse grains	1957(25.3)	5763(74.7)		
Unidentified	801(24.7)	2447(75.3)		
Food taste preference				
Light	1774(25.2)	5267(74.8)	3.728	0.155
Salty	2450(26.4)	6820(73.6)		
Unidentified	1766(25.4)	5177(74.6)		
Eating three meals on time				
< 2 times/week	1715(25.4)	5031(74.6)	1.320	0.517
2 ~ 3 times/week	1853(26.2)	5209(73.8)		
> 3 times /week	2422(25.6)	7024(74.4)		
Midnight snacks				
< 2 times/week	3299(26.6)	9084(73.4)	11.275	0.004
2 ~ 3 times/week	2372(24.9)	7168(75.1)		
> 3 times /week	319(24.0)	1012(76.0)		
Overeating				
No	5329(25.6)	15,526(74.4%)	4.503	0.034
Yes	661(27.6)	1738(72.4)		
Food Category				
Sugar-sweetened beverages				
Never	2995(26.0)	8508(74.0)	1.674	0.643
Seldom	2617(25.6)	7598(74.4)		
Sometimes	346(24.5)	1064(75.5)		
Often	32(25.4)	94(74.6)		
Coffee				
Never	4122(25.3)	12,143(74.7)	8.023	0.046
Seldom	1674(27.1)	4513(72.9)		
Sometimes	143(24.0)	454(76.0)		
Often	51(24.9)	154(75.1)		
Alcohol				
Yes	5916(25.8)	17,028(74.2)	0.586	0.444
No	74(23.9)	236(76.1)		
Milk or dairy products				
Never	2469(26.5)	6841(73.5)	5.908	0.116
Seldom	2822(25.1)	8439(74.9)		
Sometimes	554(25.9)	1583(74.1)		
Often	145(26.6)	401(73.4)		
Eggs				
Never	258(25.8)	742(74.2)	1.064	0.786
Seldom	2839(25.5)	8313(74.5)		
Sometimes	2259(26.1)	6402(73.9)		
Often	634(26.0)	1807(74.0)		
Beans or beans products				
Never	357(26.5)	991(73.5)	1.030	0.794
Seldom	3957(25.8)	11,363(74.2)		
Sometimes	1591(25.4)	4677(73.3)		
Often	85(26.7)	233(74.2)		
Fruit				
Never	441(28.7)	1096(71.3)	8.893	0.031
Seldom	3429(25.8)	9841(74.2)		
Sometimes	1795(25.1)	5356(74.9)		
Often	325(25.4)	971(74.9)		
Vegetables				
Never	1528(26.6)	4206(73.4)	7.818	0.050
Seldom	3562(25.8)	10,223(74.2)		
Sometimes	810(24.1)	2553(75.9)		
Often	90(24.2)	282(75.8)		
Livestock meat				
Never	1053(26.2)	2968(73.8)	6.838	0.077
Seldom	3426(25.2)	10,178(74.8)		
Sometimes	1363(26.7)	3743(73.3)		
Often	148(28.3)	375(71.7)		
Oil and fat				
Never	1399(25.3)	4131(74.7)	4.126	0.244
Seldom	3863(25.7)	11,183(74.3)		
Sometimes	694(27.3)	1844(72.7)		
Often	34(24.3)	106(75.7)		
Animal viscera				
Never	1129(24.0)	3581(76.0)	11.568	0.009
Seldom	4423(26.1)	12,532(73.9)		
Sometimes	432(27.6)	1134(72.4)		
Often	6(26.1)	17(73.9)		
Fish and shrimp				
Never	188(24.0)	596(76.0)	2.253	0.522
Seldom	4100(25.7)	11,843(74.3)		
Sometimes	1664(26.1)	4701(73.9)		
Often	38(23.5)	124(76.5)		

Notes: “never”, “seldom (1 ~ 2 times/week)”, “sometimes (3 ~ 5 times/week)” and “often (> 5 times/week)”

### Prevalence of *Helicobacter pylori* infection, exercise, and sleep

Table 3 showed that whether participants exercised regularly or had any one of the four sleeping problems had no significant effect on *H. pylori *infection (all P values > 0.05). There was no significant difference in *H. pylori* infection among participants with different nighttime sleep durations (*P* = 0.093). Among regularly exercising people, exercising 3 ~ 5 times/week showed the lowest rate of *H. pylori *infection (*p* = 0.039).

**Table 3:** Correlation between the prevalence of *Helicobacter pylori* infection, exercise, and sleep

Variable	*H. pylori*-positive *N*(%)	*H. pylori*-negative *N*(%)	X ^2^	*P* values
Exercise regularly				
Yes	3814(25.5)	11,117(74.5)	1.007	0.316
No	2176(26.1)	6147(73.9)		
Exercise time				
< 5 years	1334(26.1)	3777(73.9)	4.130	0.389
6 ~ 10 years	454(25.1)	1355(74.9)		
11 ~ 15years	1018(24.5)	3137(75.5)		
16–20 years	413(26.1)	1171(73.9)		
≥ 21years	595(26.2)	1677(73.8)		
Frequency of exercise per week				
1 ~ 2 times	1659(25.7)	4788(74.3)	6.506	0.039
3 ~ 5 times	1423(24.6)	4364(75.4)		
> 5 times	732(27.1)	1965(72.9)		
Sustained exercise time per day				
< 30 min	883(26.9)	2394(73.1)	4.742	0.093
30 ~ 60 min	2176(25.0)	6528(75.0)		
> 60 min	755(25.6)	2195(74.4)		
Night sleeping time				
< 5 h	686(27.6)	1798(72.4)	6.411	0.093
5 ~ 7 h	4054(25.3)	11,943(74.7)		
8 ~ 9 h	1209(26.2)	3405(73.8)		
> 9 h	41(25.8)	118(74.2)		
Difficulty falling asleep				
Yes	1000(26.5)	2772(73.5)	1.332	0.248
No	4990(25.6)	14,492(74.4)		
Early-wake				
Yes	962(26.9)	2618(73.1)	2.739	0.098
No	5028(25.6)	14,646(74.4)		
Dreaminess or awake from nightmare				
Yes	638(26.7)	1749(73.3)	1.306	0.253
No	5352(25.6)	15,515(74.4)		
Nighttime awakening				
Yes	735(26.3)	2065(73.8)	0.401	0.526
No	5255(25.7)	15,199(74.2)		

### Multivariate analysis of the correlation between risk factors and *H. pylori* infection

The total test of model coefficients χ^2^ = 223.695, *p* < 0.001, and the logistic regression equation had statistical significance. Participants’ education level (OR: 0.911; 95%CI: 0.881-0942) was a protective factor against* H. pylori* infection. Participants’ marital status (divorced or married) (OR: 1.16; 95%CI: 1.090–1.234) was a risk factor for *H. pylori* infection. In lifestyle factors, exercise and sleep showed no significant relationship with* H. pylori* infection. Participants’ diet patterns showed a significant relationship with *H.pylori* infection. Overeating (OR: 1.105; 95%CI: 1.001–1.220) and egg (OR: 1.047; 95%CI: 1.004–1.092), animal viscera (OR: 1.077; 95%CI: 1.014–1.144), and coffee consumption (OR: 1.074; 95%CI: 1.019–1.132) were risk factors for *H. pylori* infection. Consumption of midnight snacks (OR: 0.926; 95%CI: 0.877–0.977) and vegetables (OR: 0.927; 95%CI: 0.884–0.972) was a protective factor for *H. pylori* infection. Details are in Table 4.

**Table 4:** Risk factors associated with the prevalence of *Helicobacter pylori*

Variable	Coefficient	*P* value	OR	95% C.I.	
Educational background	-0.093	**< 0.001**	0.911	0.881	0.942
Marital status	0.148	**< 0.001**	1.160	1.090	1.234
Occupation Student		**< 0.001**			
Unemployed	1.129	**0.005**	3.093	1.400	6.837
Farmer	1.107	**0.007**	3.025	1.361	6.720
Professional and technical personnel	1.061	**0.008**	2.891	1.320	6.328
Official	1.108	**0.006**	3.027	1.381	6.636
Business manager	1.261	**0.002**	3.529	1.611	7.732
Factory worker	1.091	**0.008**	2.978	1.334	6.652
Retired people	1.055	**0.010**	2.873	1.294	6.376
Other	0.971	**0.015**	2.642	1.203	5.800
Eating three meals on time	0.032	0.081	1.033	0.996	1.071
Midnight snack	-0.077	**0.005**	0.926	0.877	0.977
Overeating	0.100	**0.047**	1.105	1.001	1.220
Staple food	-0.025	0.134	0.976	0.945	1.008
Eggs	0.046	**0.032**	1.047	1.004	1.092
Fruit	-0.040	0.085	0.960	0.917	1.006
Vegetable	-0.076	**0.002**	0.927	0.884	0.972
Livestock meat	0.042	0.069	1.042	0.997	1.090
Animal viscera	0.074	**0.015**	1.077	1.014	1.144
Coffee	0.071	**0.007**	1.074	1.019	1.132
Constant	-2.282	< 0.001	0.102		

### Diet pattern related to *H. pylori* infection in different occupations

**Fig. 2:**
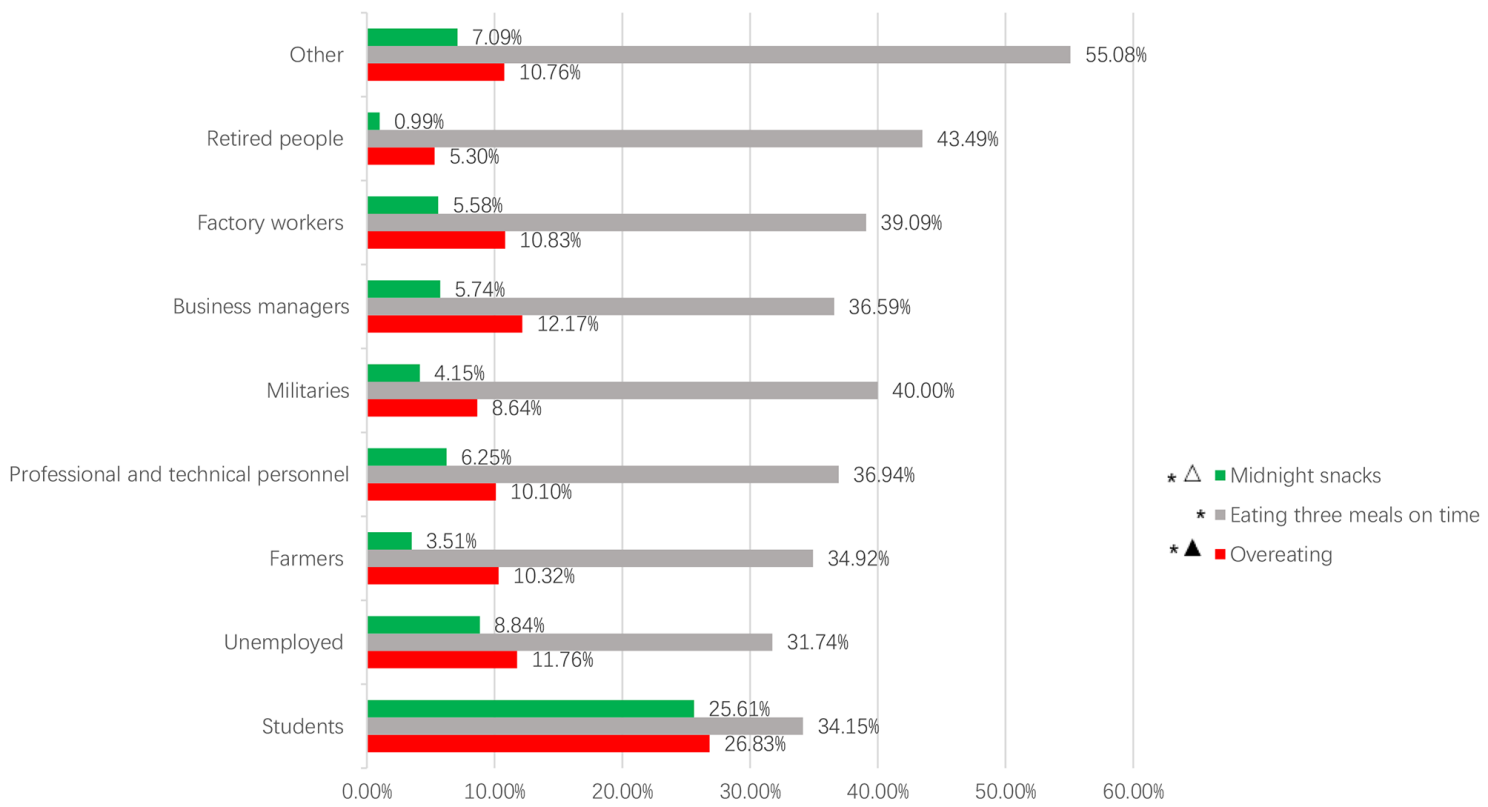
The percentage of eating meals on time, midnight snacks, and overeating more than 3 times/week ▲: risk factor for *H. pylori* infection △: protective factor for *H. pylori* infection *: P values < 0.05, comparison between occupations

According to the results of multivariate analysis, diet patterns related to *H. pylori *infection in different occupations are shown in Fig. 2. Figure 2 revealed that more than 30% of participants reported eating three meals on time > 3 times/week, and less than 15% of participants reported overeating and taking midnight snacks > 3 times/week. Participants from different professions showed significant differences in the rates of overeating, eating three meals on time, and midnight snack consumption > 3 times/week (P values < 0.05). Students had the highest high-frequency (> 3 times/week) midnight snacking and overeating per week, and retired people had the lowest proportion.

**Fig. 3:**
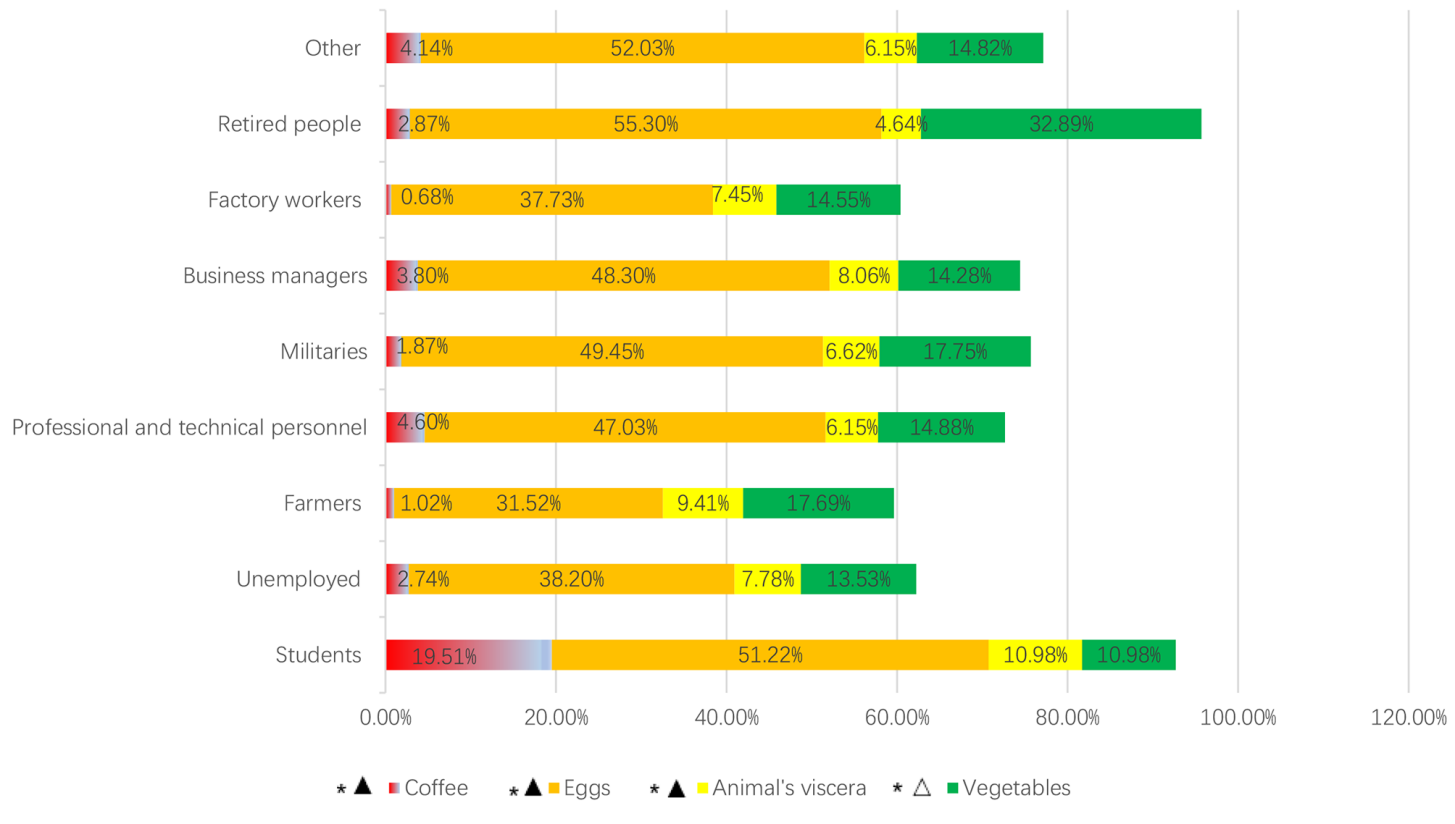
The percentage of intaking coffee, eggs, animal’s viscera and vegetables more than 3 times/week Notes: ▲: risk factor for *H. pylori* infection △: protective factor for *H. pylori* infection *: P values < 0.05, comparison between occupations

Figure 3 revealed that more than 30% of participants reported egg consumption > 3 times/week, and less than 30% of participants reported vegetable consumption > 3 times/week. Participants with different professions showed significant differences in the rates of consuming coffee, eggs, animal viscera, and vegetables > 3 times/week (P values < 0.05). Students had the highest proportion of high-frequency risk foods related to *H. pylori *infection, and farmers had the lowest proportion.

## Discussion

This study showed that the overall *H. pylori* infection rate in the Hunan cohort of central China was 25.8%, which is similar to that in the Chongqing cohort of central China (24.58%) [28] and lower than that in the Zhengzhou cohort of central China (54.27%) [29]. This reflected that the prevention and control of *H. pylori* infection in Hunan areas has a good effect. With efforts to prevent and control *H. pylori*, such as a family-based *H. pylori* prevention and eradication strategy (2021 edition), the infection rate has decreased over the past decades [10, 30]. On the other hand, the difference in the monitoring methods of *H. pylori* in different regions may cause conflicting results regarding the *H. pylori* infection rate.

This study reported that marital status was significantly correlated with *H. pylori*, but gender, chronic disease, and medical insurance were not. These results were consistent with those of previous studies [4, 31]. The higher the education and socioeconomic level of the people, the stronger their awareness of preventing *H. pylori* infection [4, 15, 16]. Previous studies showed that the *H. pylori* infection rate was higher in married couples than in single individuals [31] and increased with the duration of marriage [29]. Both partners could be infected (34.84%) and share the same strains (34.55%) [16, 29].

A regular meal pattern includes meal frequency and meal time, and “eating three meals per day” is the typical practice [32]. This study reported that eating three meals on time had no significant effect on *H. pylori* infection. Still, overeating was a risk factor for *H. pylori* infection. Regular fasting periods could reduce inflammation and modulate the gut microbiota related to *H. pylori* infection [32]. People who deviated from their meals by 2 h or more, twice or more per week, were six times more likely to have *H. pylori* infection with gastritis [33]. Overeating and midnight snacks break the regular meal pattern. Overeating and snacking were risk factors for *H. pylori* infection, and they changed gut microflora, resulting in dysbiosis, increased intestinal permeability, and leakage of toxic bacterial metabolites into the circulation, all of which contribute to the development of low-grade systemic inflammation [34, 35].

Differently, participants who consumed midnight snacks > 3 times/week showed a lower rate of *H. pylori *infection in this study. One of the reasons may be that people with a high frequency of midnight snacks had a higher percentage of insufficient dietary diversity [36], which means that people have a lower chance of consuming food susceptible to *Helicobacter pylori* infections, such as milk, vegetables, foods of animal origin, and ready-to-eat foods [37]. Besides, this cross-sectional study may lead to a contrary relationship to our traditional belief that restricting late-night snacks is more beneficial for health. Anyway, meal patterns had some effect on *H. pylori* infection.

This study indicated that coffee was a risk factor for* H. pylori *infection, and sugar-sweetened beverages and alcohol showed no significant correlation with *H. pylori* infection, corresponding to previous studies [8, 16, 38]. In contrast, Habbash and colleagues indicated that green tea, honey, and coffee were independent protectors against *H. pylori *infection [15]. This may be because coffee and tea contributed the most to the dietary total antioxidant capacity, which was associated with a reduced risk of *H. pylori* infection in adults [39, 40]. For people with active *H. pylori* infection, coffee was positively related to upper gastrointestinal symptoms [41], and coffee drinkers had worse symptom improvement after eradication therapy [42, 43]. For alcohol drinkers, some studies have shown that alcohol consumption is a risk factor for *H. pylori* infection, and binge drinking is positively associated with *H. pylori* infection [13, 43]. An intake of 10, 15, 30, 60, and 96 g/day alcohol could reduce *H. pylori* infection by approximately 22% and may facilitate the elimination of *H. pylori* [44]. Therefore, more research is needed on the relationship between coffee and alcohol and *H. pylori* infections, especially in frequency and amount of consumption.

Vegetables were a protective factor against *H. pylori* infection [8, 45], while animal viscera and eggs could increase the risk of *H. pylori* infection in this study. *H. pylori* infection is associated with metabolic disorders, and *H. pylori* infection may exacerbate the dysbiosis of the gut microenvironment induced by a high-fat diet, including alterations in the microbiota and metabolites [46]. Vegetable extracts (Broccoli sprouts, curcumin, Burdock complex, and Nigella sativa) and micronutrients (vitamins C and E) synergized with pharmacological therapies to eradicate *H. pylori* but showed a slight effect on clearing *H. pylori* as single agents [47]. Thus, vegetable consumption was encouraged to decrease the risk of *H.pylori* infection.

Milk or dairy products, beans or bean products, livestock meat, oil and fat, and seafood had no significant correlation with *H. pylori* infection in this study, same as previous studies [32, 45]. A meta-analysis showed that intake of these foods could reduce the incidence of *H. pylori *infection [11]. Chicken egg yolk, which contains immunoglobulin Y (IgY), could minimize harm from animal sources without inducing specific resistance, and thus it has been an alternative treatment for *H. pylori* infection [48]. Bovine milk could resist *H. pylori* by turning caseins into cascading 17 and β-casein 207–224 under pepsin [49]. However, *H. pylori* exist in food: raw milk from bovines (7.5%), ovines (17.27%), caprines (13.84%), buffaloes (10.76%), camels (5%), and 12% of shellfish [50, 51]. Quaglia and colleagues thought that the intake of seawater, milk, foods of animal origin, and ready-to-eat foods could increase the chance of *H. pylori* infection [37]. Therefore, future studies should control for cooking methods, food quality, and hygiene factors when analyzing the correlation between food and *H. pylori* infection.

Few studies reported the correlation between physical activity, sleep, and* H. pylori* infection. Like previous study [15], physical activity and sleep showed no significant correlation with *H. pylori* infection. Most studies revealed the correlation between exercise, sleep disorders, and gastric disease caused by *H. pylori *infection. Regular exercise reduces the risk of gastric cancer related to *H. pylori *infection [8, 17]. Moderate physical activity could reduce gastric secretions, enhance immune function, reduce anxiety, and encourage the adoption of a healthy lifestyle. Prolonged endurance exercise might suppress immune function and mucosal blood flow [51]. Sleep disorders could increase the risk of ulcers caused by* H. pylori* infection. Poor sleepers were more likely to have peptic ulcer disease or peptic ulcer disease recurrence, especially sleepers who experienced longer sleep onset latency and more nighttime awakenings [19, 52, 53]. During deeper sleep stages, the human body increases defensive factors (gastric bicarbonate efflux, gastric mucosal blood flow, and melatonin secretion) and decreases aggressive mediators (gastrin secretion) against gastrointestinal diseases [18, 19]. Therefore, physical activity and sleep seem to have a minor effect on *H. pylori* infection and more evidence was needed.

This study revealed that business managers, farmers, and the unemployed had the highest infection rates, while students, professional and technical personnel, and militaries reported the lowest. Business managers were more likely to be infected with *H. pylori* through face-to-face communication or close physical proximity when conducting business activities, such as producing or servicing [54]. Farmers showed a significantly higher *H. pylori* infection rate than nonfarmers and had the highest* H. pylori* prevalence among agricultural, forestry, and fishery workers [4, 55]. Farm animals increase the *Helicobacter pylori* infection in farmers, with pets and farm animals could transmit *H. pylori* infection to humans [56]. Elhariri and colleagues also reported that the milk and feces from healthy cattle, buffaloes, and sheep were the shedding site of *H. pylori *(especially the virulent *H. pylori* strains), and feces showed as a possible source of milk contamination [57]. Students and professional and technical personnel had more knowledge about *H. pylori*, which may lead to a lower *H. pylori *infection rate [43]. A lower prevalence of infection in the military may be because most soldiers are young males and are typically healthier than the general population [55]. The three meals provided to students and militaries had strict requirements, which may also be a related factor. As a result, business managers, farmers, and unemployed people need to improve the prevention of *H. pylori* infection.

Occupational status was one of the factors associated with *H. pylori* infection [58], but the benefit of eradication at the occupational level has not yet been adequately studied [23]. This study supported that the prevalence of *H. pylori* infection between different occupations was significantly different, and different occupations showed different dietary patterns. The prevalence of *H. pylori* infection has greatly decreased in adults, but not children and adolescents in any World Health Organization regions during the last three decades [5]. This study showed that dietary patterns in children showed a high risk of *H. pylori* infection, and the *H. pylori *infection rate will increase with students’ graduation and joining the workforce if their dietary patterns are not changed. Decreasing the frequency of overeating and midnight snacks, reducing the consumption of eggs, animal viscera, and coffee, and increasing vegetable consumption was urgent for students. For farmers, factory workers, and unemployed people, although their high frequency (> 3 times/week) of coffee and egg consumption was relatively low, reducing the frequency of animal viscera consumption and overeating was supported. Retired people, militaries, and professional and technical personnel showed good control of animal viscera and coffee consumption, but not egg consumption. Business managers should limit the consumption of coffee and animal viscera.

Above all, this article reveals that different occupational practitioners have different dietary patterns, and dietary patterns have a certain correlation with *Helicobacter pylori* infection. This may be related to the nutritional elements in the food, the number of *Helicobacter pylori* infections in the food, and the cooking method of the food. Therefore, studying and setting up occupation-related measures for *Helicobacter pylori* prevention should be noticed in the future.

### Strengths and limitations

As we know, this is the first study to combine diet, exercise, and sleep factors to analyze the impact of lifestyle on *H. pylori* infection in different occupations. This study provided a preliminary *H. pylori* infection prevention at the occupational level. On the side of lifestyle, this study explained the poor control of *Helicobacter pylori* infection in adolescents and children to a certain degree. There were some limitations in this study. First, this study included participants undergoing a physical examination, which has to some extent overlooked the portion of the population with poor awareness of self-health management. Second, a cross-sectional study cannot reflect the time trend of *H. pylori* infection. Finally, selection bias needs to be considered. The samples for this study mainly come from the physical examination population in Hunan areas, which may cause the samples to be homogeneous. Finally, living in the same house with *H. pylori*-positive relatives was more likely to result in an *H. pylori* infection [14]. It is necessary to analyze the relationship between eating habits and *H. pylori* infection after controlling for residents in the same house in future research.

## Conclusions

This study indicated that the overall *H. pylori* infection rate in the Hunan area was 25.8%. People who were business managers, farmers, or unemployed showed the highest rate of *H. pylori* infection. Although this study reveals that independent eating habits, frequency of food consumption, exercise, and sleep have almost no effect on *H. pylori* infection, after combining the occupational and social background, the diet pattern affects *H. pylori* infection significantly. Thus, people need to avoid overeating (especially for students and business managers), coffee (especially for students and professional and technical personnel), and animal viscera (especially for students and farmers), increase the intake of vegetables (especially for students and unemployed people). These findings provide new insights into public preventive measures for *H. pylori *infection and outline the areas of potential focus.

## Data Availability

The datasets used and/or analyzed during the current study are available from the corresponding author on reasonable request.
